# Machine learning to predict hospital admission at triage in paediatric emergency care: A meta-analysis

**DOI:** 10.1007/s00431-026-06895-6

**Published:** 2026-03-31

**Authors:** Blanca Paola Pérez, Octavio Galindo Osorio, Mónica Arias-Colinas, José Manuel Moreno, Nerea Martín-Calvo

**Affiliations:** 1 School of Medicine, Department of Preventive Medicine and Public Health, University of Navarra, 31008 Pamplona, Spain; 2 Department of Pediatrics, Clinica Universidad Navarra, Madrid, Spain; 3 IdiSNa (Medical Research Institute of Navarra), Pamplona, Spain; 4 CIBER Physiopathology of Obesity and Nutrition, Carlos III Health Institute, Madrid, Spain; 5 School of Nursing, Department of Nursing Care for Adult Patients, University of Navarra, Campus Universitario, 31008 Pamplona, Spain

**Keywords:** Machine learning, Deep learning, Paediatric emergency department, Triage, Hospitalization prediction

## Abstract

Machine learning (ML) models have shown promise improving outcome prediction and early risk stratification in paediatric emergency department (ED) triage. This review aims to evaluate the diagnostic performance of ML in predicting hospital admissions from the data collected at triage in paediatric emergency departments (EDs). Searches were conducted in PubMed, Ovid, Scopus, and Web of Science. Two reviewers screened 264 abstracts after duplicate removal, excluding 239 not meeting inclusion criteria. Of the 25 full-texts assessed, 15 were excluded for outcome mismatch, leaving 10 for data extraction. Data were thereafter extracted including population characteristics, ML methods, and diagnostic metrics: area under the curve (AUC), sensitivity, and specificity. Most studies used retrospective cohorts from electronic records or national databases. Sample sizes ranged from 9,069 to over 2.9 million. AUCs ranged from 0.78 to 0.97, with top-performing models (AUC ≥ 0.94) using random forest algorithms and variables like age, heart rate, triage level. Meta-analysis of six studies showed pooled sensitivity of 0.78 and specificity of 0.76 (AUC = 0.84), though heterogeneity was high (I^2^ = 100%). *Conclusion*: ML models have potential for paediatric ED triage. Standardized methods, explainable AI, and prospective validation are essential for clinical use.

**Table:** 

What is Known: • Traditional triage in paediatric emergency departments may have limitations in accurately predicting hospital admissions. • Machine learning models are increasingly applied to improve risk stratification in clinical settings.
What is New: • This review shows ML models can predict paediatric ED admissions with high AUCs (up to 0.97). • Random forest algorithms using vital signs and triage data performed best.

**Supplementary Information:**

The online version contains supplementary material available at 10.1007/s00431-026-06895-6.

## Introduction

Triage enables an initial structured patient assessment to determine clinical urgency and guide treatment prioritization. Beyond serving as the first point of contact, effective triage facilitates early identification of high-risk patients and efficient allocation of limited resources, which is essential for timely care and improved clinical outcomes [1]. Over time, paediatric emergency department (ED) visits have increased in both volume and acuity [2]. Of approximately 137 million annual ED visits in the United States, 30 million involve children [2, 3]. ED triage requires rapid clinical decisions under limited information and time. Common tools used for systematic patient assessment include the Emergency Severity Index (ESI) and Manchester Triage System (MTS) [1]. Within overloaded systems it is essential to accurately guide diagnosis and treatment from the first point of contact to improve efficiency, resource allocation and reduce negative outcomes.

Given these challenges, the integration of machine learning (ML) models into triage systems has emerged as a promising strategy to enhance early risk stratification and outcome prediction in pediatric emergency departments. Unlike conventional triage approaches based on predefined scoring rules, ML models can capture complex, nonlinear relationships among patient variables [1]. Deep learning (DL), a subset of ML, further extends these capabilities by enabling automated feature extraction and direct processing of raw, unstructured data through deep neural networks (DNNs) [4–6].

As ML increasingly informs clinical decision-making, it is essential to evaluate whether these models can be ethically and effectively integrated into ED triage workflows while preserving clinician oversight and autonomy. When applied cautiously, ML tools may complement clinical judgment by revealing previously unrecognized patterns and supporting decision-making in routine emergency care [4, 6].

Despite the expanding literature on ML applications in ED triage, no comprehensive systematic review and meta-analysis has evaluated the diagnostic performance of ML-based models applied at the point of triage in paediatric populations, with existing studies remaining fragmented due to heterogeneity in model design, data preprocessing, and evaluation metrics.

This systematic review and meta-analysis evaluate the performance of ML and DL models in predicting hospital admission among children presenting to paediatric ED using information available at the triage stage. By focusing on models developed exclusively from triage-level data, including low-dimensional approaches, we assess whether early probabilistic predictions of hospitalization and critical outcomes can provide actionable support for emergency clinicians and hospital administrators. This study does not aim to replace clinical judgment but rather to examine whether data-driven tools applied at the point of triage can complement clinical decision-making and support anticipatory resource planning in resource-constrained and overcrowded emergency care settings.

## Methods

### Literature search and selection

The Preferred Reporting Items for Systematic Reviews and Meta-Analyses (PRISMA) guidance was followed, and a review protocol was designed and registered in the international prospective register of systematic reviews (PROSPERO ID CRD42023445471).

### Inclusion and exclusion criteria

This systematic review and meta-analysis included cross-sectional and retrospective cohort studies evaluating the accuracy of at least one ML algorithm in predicting hospital admission among children presenting to paediatric EDS at the point of triage, using physician admission or discharge decisions as the reference standard. Studies focusing on alternative outcomes (e.g., ICU admission, mortality, or diagnostic testing), those in which hospital admission could not be evaluated as a discrete outcome (e.g., composite endpoints or unavailable admission-specific performance metrics), and studies including individuals over 21 years of age were excluded. Studies restricted to specific conditions were included only when hospital admission was the primary outcome.

Only studies based exclusively on triage-level data were included, as a deliberate methodological choice to ensure clinical relevance, comparability, and interpretability. Triage represents a standardized and well-defined decision-making point, enabling direct comparison with established triage systems such as the Emergency Severity Index (ESI) and the Manchester Triage System (MTS). Focusing on this early stage minimizes methodological heterogeneity and limits variability introduced by institution-specific practices, clinician behaviour, and downstream diagnostic testing. Moreover, models incorporating data obtained later in the ED course, such as laboratory or imaging results, do not reflect real-world conditions in which rapid anticipatory resource allocation is required. By restricting the analysis to this clinically meaningful time point, we aimed to reduce confounding and enhance the validity and generalizability of the synthesized evidence.

Studies were searched in the medical bibliography databases PubMed, Ovid, Scopus, and Web of Science. Searches were conducted from database inception to 25 May 2024 and there were no temporal restrictions were applied to the search strategy.

The search strategies are available in Supplementary Table 1.

**Fig. 1:**
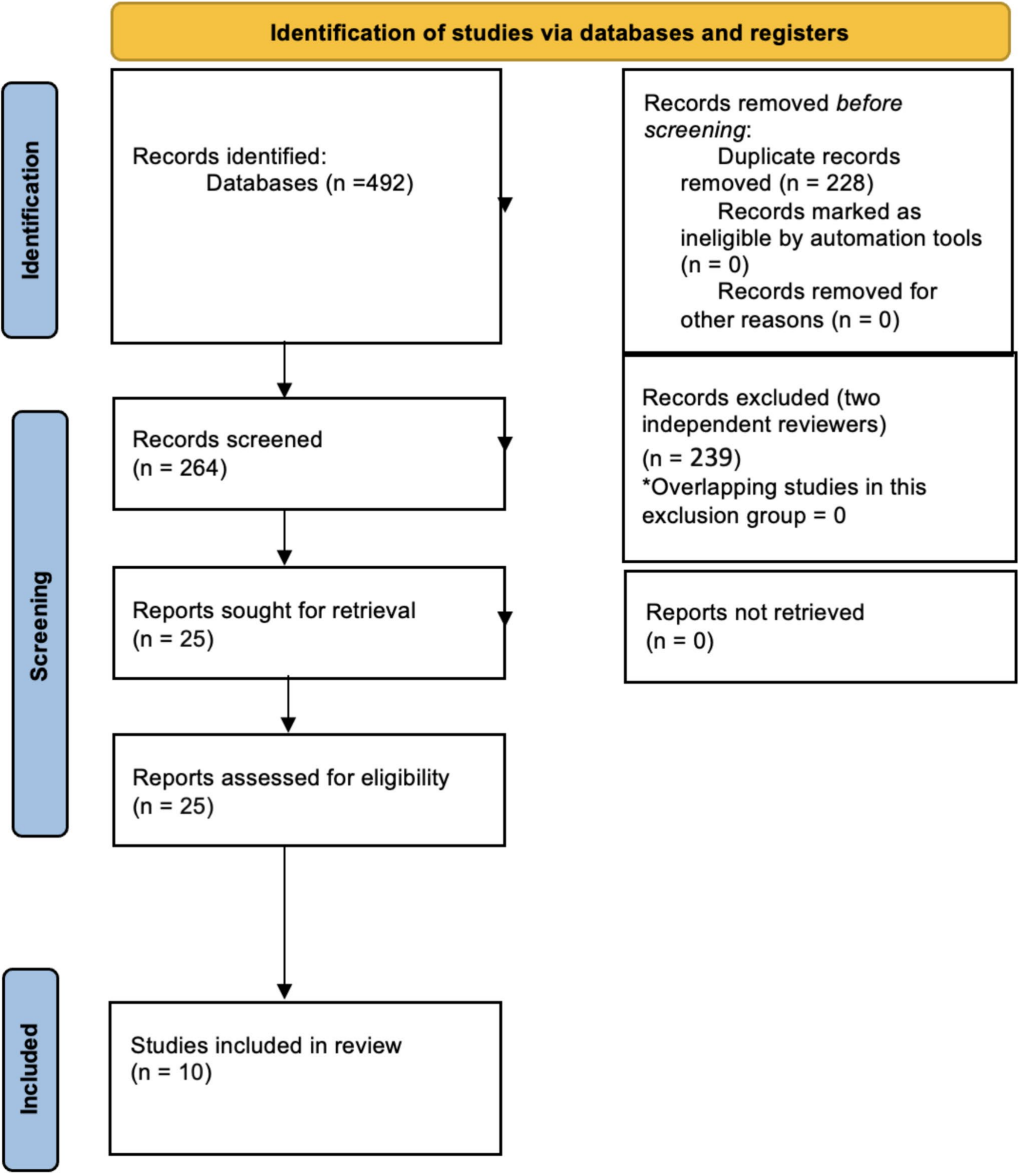
Flow diagram of the literature search and studies selection process

Two independent reviewers (BPP, NMC) conducted the search and article selection, resolving disagreements through discussion. A total of 492 records were identified, of which 228 duplicates were removed, leaving 264 records for title and abstract screening. After excluding 239 records, 25 full-text articles were assessed for eligibility. Fifteen articles were subsequently excluded, resulting in 10 studies included in the systematic review and meta-analysis. The study selection process is illustrated in Fig. 1.

In addition to the systematic database search, backward snowballing of reference lists was performed, identifying four additional records. Following full-text assessment, all were excluded for not meeting the predefined inclusion criteria, due to the absence of a paediatric population, non-cohort study design, or reliance on variables collected beyond the triage stage, thereby limiting relevance to triage-based decision-making.

### Data extraction and synthesis

Two independent reviewers (BPP, OG) extracted the relevant data from the selected articles following a standardized procedure. Extracted data included author, year of publication, country where the study was conducted, study population (sample size, age range, and sex distribution), sociodemographic factors, aims of the study, ML algorithm used, area under the curve (AUC), and diagnostic yield parameters (sensitivity, specificity, VPP, and VPN).

Hospitalization was defined as the final disposition recorded during an emergency department visit, regardless of the time elapsed from triage to that decision point.

### Quality assessment

Three independent reviewers (BPP, OG, MAC) assessed methodological quality and risk of bias using the Prediction model Risk Of Bias Assessment Tool (PROBAST) framework [7], with disagreements resolved by a fourth reviewer (NMC). Domains evaluated included patient selection, index test, reference standard, and flow and timing, along with applicability concerns related to patient selection, index test, and reference standard.

### Data analysis

Studies reporting true positive (TP), false positive (FP), true negative (TN), and false negative (FN) values—or from which these could be derived—were included in the meta-analysis. Summary sensitivity, specificity, likelihood ratios, and odds ratios were estimated using meta-analytical methods for diagnostic accuracy studies, with corresponding heterogeneity statistics. To assess robustness, sensitivity analyses were conducted excluding studies rated as high risk of bias according to the PROBAST tool.

Analyses and figures were performed using Stata version 15.0 (Stata Corp., College Station, TX, USA).

## Results

Following the full-text review, ten studies met all eligibility criteria and were included in the final assessment of the utility of ML in triage. Table 1 provides an overview of the geographical origin, study design, sample size, and demographic characteristics of the included studies. Table 2 summarizes the methodology employed in each study.

**Table 1:** Descriptive characteristics of the selected studies

	Country	Design	Information source	Uni vs. multicenter	Analyzed time period	Sample size	% HA	% Male	Age range Median age^1^ IQR^2^
Hatachi et al. (2018)	Japan	Retrospective cohort	SAKAI’s children emergency care center	Unicenter	August 2016 – October 2019	88,283	2	52.4	< 16 3.9^1^
Goto et al. (2019)	USA	Retrospective cohort	CDC National health statitics	Multicenter	January 2007 – December 2015	52,037	5	52.0	< 18 6^1^ (2–14)^2^
Wolff et al. (2919)	Chile	Retrospective cohort	Exequiel González Cortés Hospital database	Unicenter	August 2014 – October 2017	189,718	5	52.9	< 18 2.9^1^
Roquette et al. (2020)	Brazil	Retrospective cohort	Sabara Children’s hospital database	Unicenter	January 2015 – August 2018	499,853	6	52.9	< 18 2.9^1^ (1.4–5.7)^2^
Heyming et al. (2021)	USA	Retrospective cohort	EHRR Children’s Hospital Orange County California	Unicenter	March 2013—December 2020	585,142	9	53.55	< 21 4^1^
Kwon et al. (2021)	South Korea	Retrospective cohort	Korean NEDIS database	Multicenter	January 2014 – June 2016	2,937,078	13	57.4	< 15
Hwang et al. (2022)	South Korea	Cross sectional observational study	Korean NEDIS database	Multicenter	January 2016 – December 2017	262,171	12	57.2	< 15 3^1^ (1.0–7.0)^2^
Leonard et al. (2022)	Ireland	Retrospective Cohort	EHRR Data	Unicenter	January 2017 – December 2018	72,229	15	55.1	< 18
Patel et al. (2018)	USA	Retrospective Cohort	EHRR Data	Unicenter	January 2012 – December 2015	29,392	17	58	2–18
Sills et al. (2021)	USA	Retrospective Cohort	EHRR Colorado EDs	Multicenter	January 2009 – December 2013	9069	22	62.7	2–21 6^1^ (4–10)^2^

Centers for Disease Control (*CDC*), Electronic Health Record Registry (*EHRR*), Emergency Department (*ED*), Hospital Admision (*HA*), Interquartile Range (*IQR*), National Emergency Department Information System (*NEDIS*), United States of America (*USA*), and Sakai’s Children Emergency Care Center (*SAKAI*)

**Table 2:** Information on ML models used in the selected studies

Author and Date	Data pre-processing	Random assortment of data in training and testing	Validation of training sets	Models developed	Variables assessed	High discrimination variables	Diagnostic yield parameters	
Hatachi et al. (2018)	Exclusion criteria applied Handling missing values Feature engineering EHR re-labeling Encoding categorical values	No Preperiod cohort training set from August 2016 -Oct 2018 Postperiod validation cohort Oct 2018 to Oct 2019	Yes tenfold cross validation with stratification	LR SVM RF EGBx	Demographics Vital signs Triage level Chief complaints Revisit Data	Age Triage level O_2_ sat HR Revisit within 24 h	AUC-ROC	
Goto et al. (2019)	Exclusion criteria Handling missing values Feature engineering Data cleaning Encoding categorical values	Yes Random Sample training cohort Training set was 70% data set	Yes tenfold cross validation applied in training	LR with lasso regularization RF GBM DT DNN	Demographics Arrival mode Vital signs Chief Complaints Comorbidities ED revisit	Age RR O_2_ sat Arrival by ambulance Pule rate	AUC-ROC	
Wolff et al. (2019)	Data cleaning Class imbalance correction Case Re-labeling Encoding categorical values	Random stratified cross validation	Yes fivefold cross-validation Hold-out validation	RF DL NB SVM	Demographics Trauma HR, RR, Oxygen saturation BP Temp ESI Prior ED visits Pain VAS LOC	Age Sex Trauma HR ESI	AUC	
Roquette et al. (2020)	Exclusion Criteria Feature engineering Transformation of values Normalization of numerical values	No Time split Training set Jan 2015- Apr 2018 Test set May 2018-Augut 2018	Yes Train-validation split for DLM and Tree based models used 3 split time series cross validation	SVM Elastic Net DNN GBM Hybrid model (deep learning and gradient boosting)	Demographics Triage variables Vital signs Past visit data Triage text data	Triage text embeddings MTS Time interval between current and last visit O_2_ sat Age	AUROC	
Heyming et al. (2021)	Exclusion Criteria Encoding categorical value	Yes Training set 50% Validation set 10% Test set 40%	Yes 10% data was used in validation process	XGBoost	Triage vital signs: Temp, BP, HR, O2 sat and RR Nursing assessment: capillary refill, appearance, LOC, skin assessment ESI Demographics Historical Data	History of IV medication use ESI level CRT	AUROC Sensitivity PPV NPV	
Kwon et al. (2021)	Exclusion criteria Data normalization Class imbalance correction Encoding categorical values	No Derivation data Jan 2014—June 2016 Test July 2016 – December 2016	No Performance was assessed only on test set	DL RF LR	Demographics Chief complaint Symptom onset to arrival time Arrival mode Vital signs: HR, RR, Body temp, Mental status	HR Arrival mode Chief complaint RR Temp	AUROC	
Hwang et al. (2022)	Exclusion criteria Data normalization Missing value handling	Yes Data under sampling	Yes Cross validation with AUROC	RF	Demographics Vital signs Mental status Arrival mode Trauma status Time variable	Age Body temp Time from symptom onset to ED visit HR RR	AUROC	
Leonard et al. (2022)	Exclusion criteria Feature engineering	Yes Training set 70% Testing set 30%	Yes Internal validation was conducted on test set	NB LR GBM	Presenting complaint ICTS Referral source Registration month ED location Distance traveled Admission History Weekday	Presenting complaint ICTS Referral source Registration month ED location	AUC	
Patel et al. (2018)	Exclusion criteria applied Data normalization: categorical and continuous variable normalization	Yes Training set 80% Test set 20%	Yes Three-fold cross validation	DT RF Lasso LR **GBM**	DemographicSocioeconomic status SESVital signsTriage scoreWeight for ageWeather dataCommunity viral load	O_2_ sat RR Triage acuity core ESI level HR Weight for age Z score		AUROC
Sills et al. (2021)	Exclusion criteria applied Missing data handling Feature engineering	Yes	Yes AutoML framework	AutoML RF LR	DemographicTriage variablesClinical variablesTreatment variables	Prior hospitalization ESI TT Time to first medication Age		AUROC

Area Under the Curve (*AUC*), Auto Machine Learning (*AutoML*), Capillary Refill Time (*CRT*), Centers for Disease Control (*CDC*), Critical Care Outcomes (*CCO*), Decision Tree (*DT*), Deep Learning (*DL*), Deep Neural Network (*DNN*), Electronic Health Record Registry (*EHRR*), Elastic Net Regularization (*ENR*), Emergency Department (*ED*), Emergency Severity Index (*ESI*), Gradient Boosting Machines (*GB or GBM*), Heart Rate (*HR*), Intensive Care Unit (*ICU*), Irish Children’s triage system (*ICTS*), Logistic Regression (*LR*), Manchester Triage System (*MTS*), National Health Center Statistics (*NHAMCS*), Negative Predictive Value (*NPV*), Oxygen saturation (O_2_ sat) Positive Predictive Value (*PPV*), Random Forest (*RF*), Respiratory Rate (*RR*), Retrospective Cohort (*RC*), Support Vector Machine (*SVM*), Temperature (*Temp*), Time to Triage (*TT)*, True Negative (*TN*), and True Positive (*TP*)

### Quality assessment of the selected studies

The models generally aligned well with real-world clinical settings and populations, indicating high applicability (Supplementary Fig. 1). However, selection and analysis biases require cautious interpretation before clinical implementation. PROBAST assessment showed that 6 studies [8, 10–13, 16] had a high overall risk of bias, suggesting possible issues in study design, data handling, or model evaluation affecting reliability.

Despite these methodological issues, all reviewed artificial intelligence (AI) models demonstrated high clinical applicability, reinforcing their potential utility in ED triage systems. In this context, low-dimensional models are of particular interest, as they achieved AUCs comparable to those of high-dimensional models while offering superior applicability, transparency, and practicality for real-world system implementation [13].

Lastly, several models relied on clinician judgment as the gold standard for hospitalization decisions, which is problematic due to institutional variability and non-clinical influences. Only Patel et al. and Wolff et al. used standardized, objective criteria, improving reproducibility [9, 12]. Leonard et al., Sills et al. and Roquette et al. followed the ICTS, ESI and MTS, respectively—all internationally recognized—but many studies did not explain their hospitalization criteria, making comparisons between models difficult [1, 10, 13, 14].

### Most discriminatory variables

Table 3 summarizes the performance of predictive models for early risk stratification. AUCs ranged from 0.78 to 0.97, reflecting strong performance across various ML architectures. Heyming et al.’s RF model achieved the highest AUC of 0.97 (95% CI: 0.93–0.98), identifying ESI level, capillary refill, and prior intravascular medication use as key predictors [11]. Hwang et al. reported an AUC of 0.94 with RF, highlighting age, temperature, symptom onset-to-ED time, and heart rate [16]. Roquette et al.’s hybrid model (DNN + XGBoost) reached 0.89 (95% CI: 0.885–0.90), relying on MTS, age, past visits, and oxygen saturation [10]. Sills et al. used AutoML (AUC 0.91) and Hatachi et al. XGBoost (AUC 0.86), both emphasizing age, medical history, and time-sensitive ED variables like time to triage or medication [8, 14]. DL-based models by Goto and Kwon reported AUCs of 0.80 and 0.78, relying mainly on vitals (respiratory rate, pulse, oxygen saturation) and arrival modality [15, 17]. Leonard et al. disclosed an AUC of 0.853 in their GBM model, obtaining as the highest yield variables the following: presenting complaint, ICTS, referral source, registration month, ED location to predict hospitalization risk [13]. Common high-discrimination variables across studies included age (7 of 10 studies), SaO₂, heart rate, triage level/system, and respiratory rate—showing strong prognostic value.

**Table 3:** Summary of the variables with higher discrimination ability and the AUC (95% Confidence Interval (CI)) of the most discriminatory model in the selected studies

	Best model	AUC (95% CI)	High discrimination variables
Hatachi et al. (2017)	XGBoost	0.86 (0.84–0.88)	Age, triage level, oxygen saturation, heart rate, prior ED visits
Goto et al. (2019)	DNN	0.80 (0.78–0.81)	Age, respiratory rate, oxygen saturation, ambulance arrival, pulse rate
Wolff et al. (2019)	DL	0.79	Age, sex, trauma status, heart rate, ESI
Roquette et al. (2020)	Hybrid model (DNN + XGBoost)	0.89 (0.89–0.90)	Manchester triage system, age, past visits, oxygen saturation
Heyming et al. (2021)	RF	0.968 (0.967–0.969)	ESI level, capillary refill time, historical intravascular medication use
Kwon et al. (2021)	DL	0.782 (0.780–0.783)	Heart rate, arrival mode, respiratory rate, temperature
Hwang et al. (2022)	RF	0.94	Age, body temperature, symptom onset to ED time, heart rate
Leonard et al. (2022)	GBM	0.853 (0.846–0.859)	Presenting complaint, ICTS, Referral source, Registration month, ED location
Patel et al. (2018)	GBM	0.84 (0.83–0.85)	Oxygen saturation, respiratory rate, triage ESI, weight-for-age z-score
Sills et al. (2021)	AutoML	0.91	Time to triage, time to first medication, age, prior hospitalization

### Meta-analytical integration of diagnostic accuracy studies

**Fig. 2:**
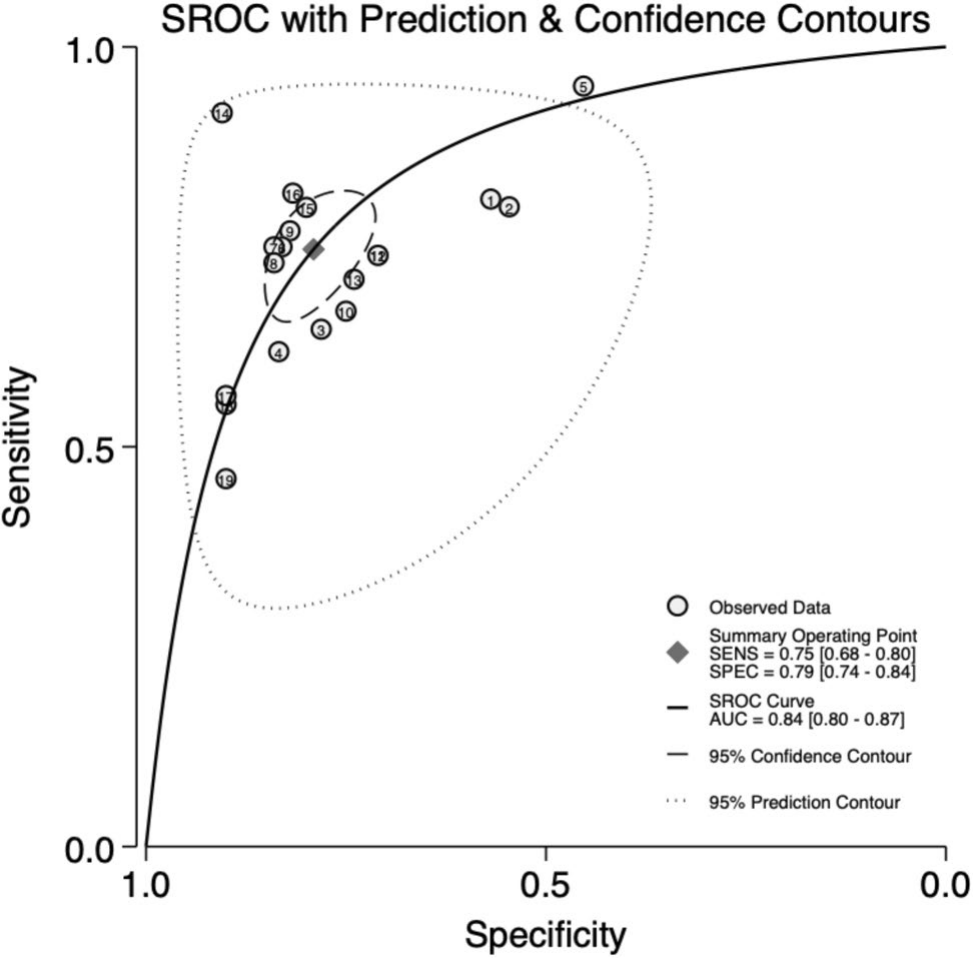
SROC with Prediction & Confidence contours of the included studies

The diagnostic performance of ML models for predicting pediatric ED hospitalizations was evaluated using a Summary Receiver Operating Characteristic (SROC) curve, as shown in Fig. 2. Studies with sufficient data to be analyzed were Wollf et al. (4 models), Patel et al. (1 model), Hatachi et al. (4 models), Goto et al. (4 models), Heyming et al. (1 model), Leonard et al. (3 models) and Roquette et al. (2 models) [8–13, 16]. Data on TP, TN, FP, and FN can be found in the supplementary Table 2. The summary operating point, represented by a red diamond, corresponds to the pooled sensitivity and specificity estimates across all included models. The summary sensitivity was 0.75 (95% CI: 0.68–0.80), indicating that the models correctly identified 75% of true hospitalization cases. The summary specificity was 0.79 (95% CI: 0.74–0.84), reflecting the ability of the models to correctly classify non-hospitalized patients in 76% of cases. Overall, these results yield that ML models exhibit high diagnostic accuracy (AUC = 0.84; 95% CI 0.80–0.87) in predicting ED hospitalizations.

The test for heterogeneity showed significant heterogeneity (p < 0.001) among studies. Furthermore, an I^2^ value of 100% suggests considerable inconsistency across studies.

The sensitivity analysis in Wolff et al. (4 models) and Goto et al. (4 models) showed similar diagnostic accuracy (AUC = 0.78; 95% CI 0.74–0.81) and heterogeneity among studies (I^2^ = 100%) (Supplementary Fig. 2) [9, 16].

## Discussion

### ML, DL and classical triage systems

This systematic review and meta-analysis synthesized findings from studies evaluating the diagnostic performance of ML models in predicting hospitalization outcomes from data collected at triage. The results indicate that ML-based triage models demonstrate high discriminative ability, with an overall AUC of 0.84 (95% CI: 0.80–0.87) (Fig. 2). This high discriminative performance has important implications for resource allocation and for alleviating overcrowding in emergency departments, particularly in underserved settings with high patient volumes. Models capable of providing an early, probabilistic estimate of admission risk before diagnostic workup is conducted may facilitate more efficient patient streaming and bed planning, thereby optimizing patient flow.

Conventional triage systems have long served as the primary tools for patient stratification and resource allocation in emergency departments. However, multiple studies have shown that machine learning–based models can achieve superior discriminative performance compared with established triage approaches such as the Korean Triage and Acuity Scale (KTAS), the ESI, and the MTS, supporting their potential role as an adjunct or alternative to traditional triage systems [11, 16, 17].

Interestingly, some included studies compared ML models with traditional triage systems. Hwang et al. found their RF model significantly outperformed the paediatric Korean Triage and Acuity Scale in predicting ICU admissions and hospitalizations, highlighting limitations of conventional systems [16]. Heyming et al. also supported ML-based triage, though their results may be unreliable due to unaddressed class imbalance [11].

Other studies compared different ML models. Patel et al. reported that their gradient-boosting machines (GBMs) had the highest predictive performance for hospitalization, reinforcing the value of ensemble-based methods in risk stratification [12]. Similarly, Hatachi et al. found GBMs performed best in their dataset but noted a tendency to over—triage, generating more false positives than expected [8]. Prioritizing sensitivity in emergency settings helps avoid missing critically ill patients, but excessive false positives can lead to unnecessary admissions, resource strain, and increased costs.

Sills et al. found that an AutoML approach improved hospitalization prediction compared to RF and LR, demonstrating the potential for automated model selection to optimize algorithmic performance without extensive manual tuning [14].

In several of the reviewed studies [9, 10, 15, 17] DL algorithms outperformed traditional triage and other ML models, suggesting they may be the most effective AI tools for evaluating complex relationships in large datasets. To provide context, ML and DL are subfields of AI. ML typically requires manual feature engineering to extract predictors, as it cannot capture complex relationships from raw inputs, while DL automates feature extraction through multi-layered DNNs and independently identifies complex patterns in unstructured data [4].

Roquette et al. was the only study to develop a hybrid ML model (DNN + XGBoost), which achieved the highest AUC (0.89, 95% CI: 0.88–0.90) among DL-based approaches. While potentially advantageous, it requires physicians to better understand how to generate and apply data for such complex models [10]. The study uniquely integrated unstructured clinical text via language normalization using DNNs, replacing the previously manual encoding needed for ML models. Surprisingly, this feature had one of the highest discriminatory capacities among all variables [10].

An outlier among DL algorithms was presented by Wolff et al., which found that RF outperformed DL algorithms and demonstrated characteristically high NPVs. High NPVs were a common trend among the models, which could be clinically significant, as they suggest that the ML model could reliably rule out critical conditions that would be useful in clinical practice, similar to the D-dimer, where a negative result could exclude diagnosis [9].

Despite promising results, these models cannot be fully relied on without external validation, as their performance on unseen data remains unknown—leaving the key ML goal of generalizability unmet. DL also raises concerns due to its low interpretability—the ‘black box effect’—making it inappropriate to base clinical judgment on algorithms that lack transparent reasoning [4, 20]. For ethical AI integration, clinicians must understand the logic behind any AI recommendations, as they remain ultimately responsible for clinical decisions [5].

There has been notable progress in the quality and transparency of ML development in paediatric ED triage, particularly through greater emphasis on data preprocessing, which is crucial for model reliability. Most studies in this review applied systematic preprocessing steps such as exclusion criteria, data normalization, categorical encoding, and handling missing data (discussed later). This marks a clear improvement over earlier research, where such steps were often omitted, limiting reproducibility and comparability of ML findings [20].

### Clinical predictors of hospital admission from triage

In asthma-related studies, respiratory rate and oxygen saturation emerged as the strongest predictors of hospitalization. In contrast, no single variable consistently predicted hospital admission across studies addressing general paediatric pathologies, likely reflecting their multifactorial nature. Instead, combinations of variables—such as clinical history, mode of transport, and vital signs including capillary refill time—were repeatedly identified as relevant predictors.

Age has been widely discussed as a fundamental parameter for predicting hospitalization across most studies. However, Leonard et al. did not include age as an input variable in their model, despite its incorporation into the Irish Children’s Triage System (ICTS) [13].

### Utility of low dimensional models

Leonard et al. demonstrate that high-performing ML models do not necessarily require high-dimensional inputs or complex architectures, as low-dimensional models achieved discrimination comparable to high-dimensional approaches (AUC 0.835 vs. 0.853) [13]. Notably, a GBM using only eight routinely collected triage variables showed strong predictive performance while preserving interpretability and ease of implementation. This is particularly relevant for settings with limited electronic infrastructure and supports the feasibility of simpler, transparent ML models over complex DL systems that contribute to the “black box” effect. Nevertheless, these findings should be interpreted with caution, as the study did not adequately address overfitting and was rated as high risk of bias under the PROBAST framework, limiting confidence in the reported results.

The advantages and disadvantages of the different ML models are described in Table 4.

**Table 4:** General advantages and disadvantages of the ML and DL models

Model	Advantages	Disadvantages
DL	- Excels at detecting complex patterns in imaging and non-structured data (EHRs and unstructured clinical narratives (e.g., anamnesis)) - Heterogeneous data handling - Improves with large data sets - Outperforms classical models in natural language processing, computer vision and EHR analysis - Minimal need for manual feature engineering	- Black box nature - Requires large and labelled datasets - Computationally expensive + + +
LR	- Simple and interpretable - Well-suited for structured data with clear linear relationships - Suitable for clinical risk prediction and binary classification tasks - Fast to train and deploy in clinical settings	- Sensitive to missing values - Struggles with complex non-linear relationships (e.g., in imaging or genomic dat
RF	- Robust to missing data - Favorable with structured tabular data - Improved interpretability - Avoids overfitting	- Computationally expensive + + - Limited performance on unstructured data such as free-text EHRs or imaging - Limited predictive power
GBM	- Strong predictive power with higher accuracy - Favorable with structured tabular data - Handles imbalanced datasets	- Prone to overfitting without careful regularization - Poor performance on unstructured data
SVM	- Favorable in small to medium datasets - Robust to avoid overfitting in small setting	- Poor performance with unstructured data - Requires extensive feature engineering and parameter tuning - Computationally expensive - Limited interpretability for clinicians
NB	- Computationally efficient for small datasets - Performs well with small sample sizes and high-dimensional data	- Requires feature independence - Performs poorly with highly correlated features

### Strengths and limitations

Our study has several strengths and limitations. One of the key strengths of this study is its rigorous methodology, adhering to PRISMA guidelines for transparency and reproducibility and employing the PROBAST framework to assess the quality and risk of bias in included studies [7]. Furthermore, this is the first systematic review and meta-analysis to evaluate the diagnostic accuracy of ML models in predicting hospital admission in the paediatric ED from data obtained at triage, providing a comprehensive synthesis of available evidence. A sensitivity analysis was conducted to assess the robustness of the findings, ensuring the reliability of the results.

Despite the promising findings, limitations must be acknowledged. The small number of included studies and the high degree of heterogeneity—stemming from variations in data preprocessing, variable selection, outcome definitions, and unspecified hospitalization timing—limit generalizability. Future meta-analyses should address these issues through study stratification or meta-regression. In addition, the absence of a standardized definition of hospital admission, often influenced by subjective clinical judgment, affects model reproducibility and warrants further investigation using more objective criteria. Furthermore, hospitalization timings were unspecified across studies and therefore will likely be a source of heterogeneity. Most models were developed using retrospective data with limited external validation, highlighting the need for prospective studies to confirm clinical utility. Incomplete reporting in some studies also impacted quality assessment. Finally, although restricting inclusion to studies using data available at triage facilitated comparability, it excluded models incorporating additional diagnostic data, potentially limiting variable identification. This was done to avoid the introduction of important disparities in training data that could lead to biased or overly optimistic estimates of diagnostic performance. Future research should incorporate a broader range of studies while accounting for differences in model development and training to support the advancement of ML implementation in paediatric emergency department triage.

## Conclusion

This systematic review and meta-analysis showed that ML and DL models demonstrate high diagnostic performance and strong discriminative ability in paediatric ED settings. However, substantial heterogeneity limits definitive conclusions, and the limited interpretability of DL models highlights the need for explainable approaches to support clinical decision-making. Despite these limitations, ML-based triage has considerable potential to improve ED efficiency, optimize resource use, and enhance paediatric patient outcomes.

## Supplementary Information

Below is the link to the electronic supplementary material.

ESM1 (DOCX 130 KB)

ESM2 (DOCX 552 KB)

ESM3 (DOCX 13.0 KB)

ESM4 (DOCX 13.5 KB)

## Data Availability

The datasets generated during and analysed during the current study are available from the corresponding author on reasonable request.

## Abbreviations

DNN: Deep neural network
ED: Emergency department
EHRs: Electronic health records
EHRR: Electronic health record registry
ENR: Elastic net regularization
ESI: Emergency severity index
XGBoost: Extreme gradient boosting machines
GBM or XGBoost: Gradient boosting machines
FN: False Negative
FP: False Positive
ICU: Intensive care unit
LR: Logistic regression
ML: Machine learning
MTS: Manchester triage system
NB: Naive Bayes
NEDIS: National emergency department information system
NHAMCS: National health centre statistics
RF: Random forest
SROC curve: Summary receiver operating characteristic
SVM: Support vector machine
TN: True Negative
TP: True Positive
