# Supplementary material for: Machine learning to predict hospital admission at triage in paediatric emergency care: A meta-analysis
Source: Eur J Pediatr. 2026 Mar 31;185(4):229. doi: 10.1007/s00431-026-06895-6 (PMC13035534; doi:10.1007/s00431-026-06895-6)

**Supplementary Figure 1. Quality assessment of the selected studies according to PROBAST (Prediction model Risk of Bias Assessment Tool)**


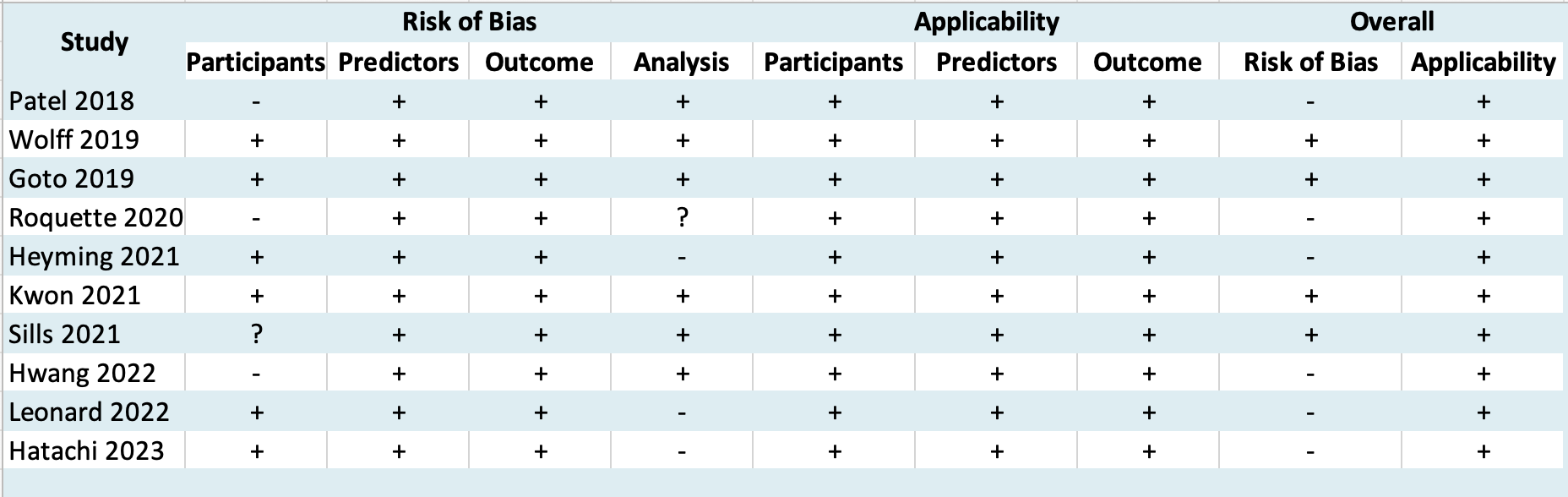

Supplement: Supplementary file 1 — (DOCX 130 KB) [file 431_2026_6895_MOESM1_ESM.docx]
