# Supplementary material for: Machine learning to predict hospital admission at triage in paediatric emergency care: A meta-analysis
Source: Eur J Pediatr. 2026 Mar 31;185(4):229. doi: 10.1007/s00431-026-06895-6 (PMC13035534; doi:10.1007/s00431-026-06895-6)

**Supplementary Figure 2. SROC with Prediction & Confidence contours after excluding the studies with high risk of bias**
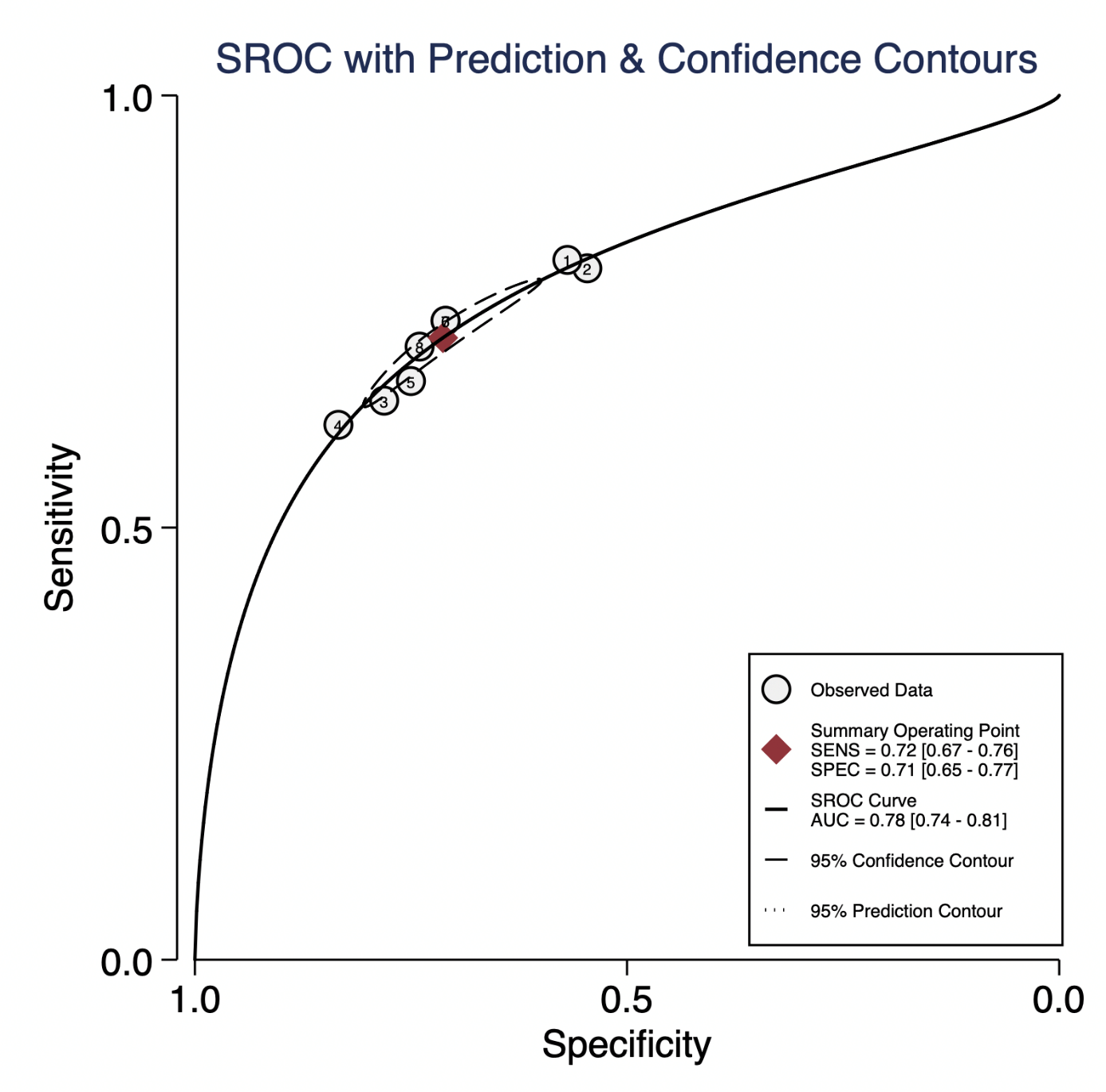

Supplement: Supplementary file 2 — (DOCX 552 KB) [file 431_2026_6895_MOESM2_ESM.docx]
