# Supplementary material for: Machine learning to predict hospital admission at triage in paediatric emergency care: A meta-analysis
Source: Eur J Pediatr. 2026 Mar 31;185(4):229. doi: 10.1007/s00431-026-06895-6 (PMC13035534; doi:10.1007/s00431-026-06895-6)
Supplement: Supplementary file 3 — (DOCX 13.0 KB) [file 431_2026_6895_MOESM3_ESM.docx]

**Supplementary Table 1. Search Strategies in databases PubMed, Ovid, Scopus, and Web of Science.**

| **Database** | **Search Strategy** |
| --- | --- |
| **Pubmed** | (((((((((pediatric) OR (pediatrics)) OR (paediatric)) OR (paediatrics)) OR (child)) OR (children)) OR ("pediatrics"[MeSH Terms])) OR ("child"[MeSH Terms])) AND ((((((triage) OR (triages)) OR (triaged)) OR (triaging)) OR ("triage"[MeSH Terms])) AND ((((((((((((((((("Artificial intelligence") OR ("Machine Learning")) OR ("Prediction model")) OR ("Predictive model")) OR ("Random Forest")) OR ("XGBoost")) OR ("Boosting Machine Learning Algorithms")) OR ("Gradient boosting")) OR ("Neural network")) OR ("Neural Networks, Computer")) OR ("Deep learning")) OR ("artificial intelligence"[MeSH Terms])) OR ("machine learning"[MeSH Terms])) OR ("predictive learning models"[MeSH Terms])) OR ("random forest"[MeSH Terms])) OR ("boosting machine learning algorithms"[MeSH Terms])) OR ("neural networks, computer"[MeSH Terms])))) AND (((((((disposition) OR ("admission prediction")) OR (hospitalization)) OR ("hospital admission")) OR ("risk prediction")) OR ("risk stratification")) OR ("hospitalization"[MeSH Terms])) |
| **Scopus** | TITLE-ABS-KEY (pediatric OR pediatrics OR paediatric OR paediatrics OR child OR children) AND TITLE-ABS-KEY (triage OR triages OR triaged OR triaging) AND  TITLE-ABS-KEY ("artificial intelligence" OR "machine learning" OR "prediction model" OR "predictive model" OR "predictive learning models" OR "random forest" OR xgboost OR "boosting machine learning algorithms" OR "gradient boosting" OR "neural network" OR "deep learning") AND TITLE-ABS-KEY (disposition OR "admission prediction" OR hospitalization OR "hospital admission" OR "risk prediction" OR "risk stratification") |
| **Web of Science** | (((TS=(pediatric)) AND TS=(triage)) AND TS=(artificial intelligence OR "machine learning" OR "prediction model" OR "predictive model" OR "predictive learning models" OR "random forest" OR xgboost OR "boosting machine learning algorithms" OR "gradient boosting" OR "neural network" OR "deep learning")) AND TS=(hospitalization OR "admission prediction" OR hospitalization OR "hospital admission" OR "risk prediction" OR "risk stratification") |
| **Ovid** | (exp Pediatrics/ OR exp Child/ OR pediatric.mp.) AND (exp Triage/ OR triage.mp. OR triages.mp. OR triaged.mp. OR triaging.mp.) AND (exp Artificial Intelligence/ OR exp Machine Learning/ OR exp Neural Networks, Computer/ OR exp Random Forest/ OR artificial intelligence.mp. OR machine learning.mp. OR prediction model.mp. OR predictive model.mp. OR predictive learning models.mp. OR random forest.mp. OR xgboost.mp. OR boosting machine learning algorithms.mp. OR gradient boosting.mp. OR neural network.mp. OR deep learning.mp.) AND (exp Hospitalization/ OR disposition.mp. OR admission prediction.mp. OR hospitalization.mp. OR hospital admission.mp. OR risk prediction.mp. OR risk stratification.mp.) |

Searches were conducted from database inception to 25 May 2024.
