# Supplementary material for: Machine learning to predict hospital admission at triage in paediatric emergency care: A meta-analysis
Source: Eur J Pediatr. 2026 Mar 31;185(4):229. doi: 10.1007/s00431-026-06895-6 (PMC13035534; doi:10.1007/s00431-026-06895-6)
Supplement: Supplementary file 4 — (DOCX 13.5 KB) [file 431_2026_6895_MOESM4_ESM.docx]

**Supplementary Table 2. True Positive (TP), True Negative (TN), False Positive (FP and False Negative (FN) of the models included in the meta-analysis.**

| **Study** | **Model** | **TP** | **TN** | **FP** | **FN** | **N** | **admission** |
| --- | --- | --- | --- | --- | --- | --- | --- |
| Wolff | DNN | 1502 | 20534 | 15554 | 353 | 37944 | 1855 |
| Wolff | DNN | 1484 | 19704 | 16384 | 371 | 37944 | 1855 |
| Wolff | NB | 1200 | 28186 | 7903 | 655 | 37944 | 1855 |
| Wolff | RF | 1148 | 30098 | 5991 | 707 | 37944 | 1855 |
| Patel | GB | 950 | 2210 | 2668 | 49 | 5878 | 999 |
| Hatachi | LR | 489 | 23422 | 4797 | 163 | 28871 | 652 |
| Hatachi | SVM | 489 | 23704 | 4515 | 163 | 28871 | 652 |
| Hatachi | RF | 476 | 23704 | 4515 | 176 | 28871 | 652 |
| Hatachi | GB | 502 | 23139 | 5079 | 150 | 28871 | 652 |
| Goto | LR | 470 | 11181 | 3727 | 232 | 15611 | 702 |
| Goto | RF | 519 | 10585 | 4323 | 183 | 15611 | 702 |
| Goto | GB | 519 | 10585 | 4323 | 183 | 15611 | 702 |
| Goto | DNN | 498 | 11032 | 3876 | 204 | 15611 | 702 |
| Heyming | GB | 15271 | 196355 | 20665 | 1371 | 233662 | 16642 |
| Roquette | GB | 1617 | 24193 | 6066 | 406 | 32282 | 2023 |
| Roquette | GB | 1653 | 24704 | 5555 | 370 | 32282 | 2023 |
| Leonard | GB | 1932 | 17948 | 1994 | 1494 | 23368 | 3426 |
| Leonard | LR | 1894 | 17947 | 1995 | 1532 | 23368 | 3426 |
| Leonard | NB | 1576 | 17948 | 1994 | 1850 | 23368 | 3426 |
